# Interaction of 6-Thioguanine with Aluminum Metal–Organic Framework Assisted by Mechano-Chemistry, In Vitro Delayed Drug Release, and Time-Dependent Toxicity to Leukemia Cells

**DOI:** 10.3390/nano14191571

**Published:** 2024-09-29

**Authors:** Sheriff Umar, Xavier Welch, Chihurumanya Obichere, Brandon Carter-Cooper, Alexander Samokhvalov

**Affiliations:** 1 Department of Chemistry, Morgan State University, 1700 East Cold Spring Lane, Baltimore, MD 21251, USA; 2 Translational Laboratory Shared Services (TLSS), The University of Maryland School of Medicine’s & Greenebaum Comprehensive Cancer Center, 22 S. Greene Street, Baltimore, MD 21201, USA

**Keywords:** leukemia, cancer, 6-thioguanine, metal–organic framework, delayed drug release, mechano-chemistry

## Abstract

6-thioguanine (6-TG) is an antimetabolite drug of purine structure, approved by the FDA for the treatment of acute myeloid lesukemia, and it is of interest in treating other diseases. The interaction of drugs with matrices is of interest to achieving a delayed, sustained, and local release. The interaction of 6-TG with an aluminum metal–organic framework (Al-MOF) DUT-4 is studied using a novel experimental approach, namely, mechano-chemistry by liquid-assisted grinding (LAG). The bonding of 6-TG to the DUT-4 matrix in the composite (6-TG)(DUT-4) was studied using ATR-FTIR spectroscopy and XRD. This interaction involves amino groups and C and N atoms of the heterocyclic ring of 6-TG, as well as the carboxylate COO^−^ and (Al)O-H groups of the matrix, indicating the formation of the complex. Next, an in vitro delayed release of 6-TG was studied from composite powder versus pure 6-TG in phosphate buffered saline (PBS) at 37 °C. Herein, an automated drug dissolution apparatus with an autosampler was utilized, and the molar concentration of the released 6-TG was determined using an HPLC–UV analysis. Pure 6-TG shows a quick (<300 min) dissolution, while the composite gives the dissolution of non-bonded 6-TG, followed by a significantly (factor 6) slower release of the bonded drug. Each step of the release follows the kinetic pseudo-first-order rate law with distinct rate constants. Then, a pharmaceutical shaped body was prepared from the composite, and it yields a significantly delayed release of 6-TG for up to 10 days; a sustained release is observed with the 6-TG concentration being within the therapeutically relevant window. Finally, the composite shows a time-dependent (up to 9 days) stronger inhibition of leukemia MV-4-11 cell colonies than 6-TG.

## 1. Introduction

**Figure 1:**
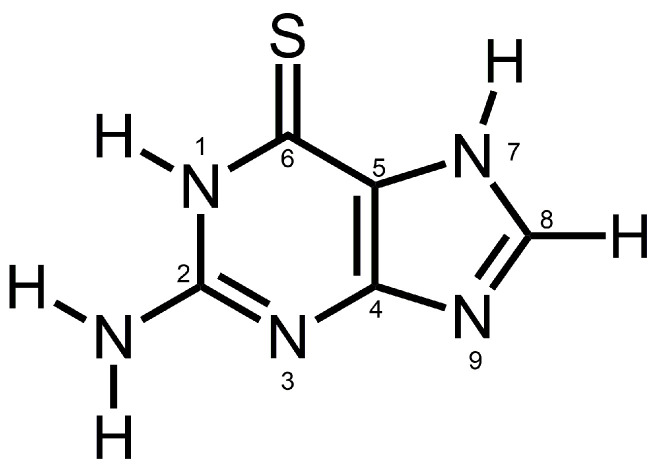
The molecular structure of 6-thioguanine (6-TG).

Leukemia is a blood cancer with high mortality; specifically, acute myeloid leukemia (AML) [1] progresses very quickly, with a 5-year survival rate of only 26%. 6-thioguanine (6-TG), shown in Figure 1, is an antimetabolite of purine structure, approved by the FDA for the treatment of AML as the TABLOID^®^ formulation, which is also studied for the treatment of other leukemias [2].

Beyond leukemia, 6-TG has been studied for treating colorectal carcinoma [3] and it has potential in treating other cancers [4] and non-cancer diseases [5]. When administered orally (systemically), 6-TG has a rather short median plasma half-life of ca. 80 min, and shows significant side effects due to the “burst”—a quick increase in the concentration of the drug in the bodily fluids [6]. To avoid the “burst”, a periodic, time-delayed, and local release can be utilized. The available methods for the controlled release of 6-TG are by using totally implanted vascular access devices (TIVADs), which are periodically inserted to release a solution of the pure drug to the blood [7].

For a continuous and delayed drug release, suitable matrices are utilized to form the pharmaceutical composites “Matrix/Drug”. Due to a simple purine molecular structure resembling many other drugs or their major functional groups, 6-TG can serve as the archetypal antimetabolite in studies of delayed release. The “Matrix/Drug” composites can be in a soluble (nano-colloid) or insoluble (e.g., powder) form. There are only very few reports of the interactions of 6-TG with matrices for drug delivery [8] and they were conducted with nano-colloids only. There are no reports of the delayed release of 6-TG from the composite with any solid-state water-insoluble matrix.

Metal–organic frameworks (MOFs) are 3D coordination polymers, which consist of structure-building organic linkers and metal cations. MOFs are efficient matrices for “guests”, specifically drug molecules [9], which results in a delayed release [10] and minimization of the “burst”. MOFs can be nano-colloidal [11] or water-insoluble (powder) matrices [12]. Aluminum MOFs, Al–MOFs [13], are insoluble in any solvent, stable in aqueous solutions, and have low cytotoxicity [14]. This makes them a promising platform for delayed drug release via drug-eluting implants [15]. Surprisingly, studies of the interactions of biologically active compounds with Al–MOFs and their delayed release are rare. Nakahama et al. [16] reported the interaction with encapsulation and the delayed release of L-glutamic acid by the three isoreticular Al–MOFs. There are no reports of the delayed release of 6-TG from composites with any MOFs.

Usually, encapsulation is performed by the sorption of drug molecules from a solution on a matrices including MOFs [17]. Unfortunately, many antimetabolites of purine structure (e.g., 6-TG), as well as of pyrimidine structure (e.g., gemcitabine), have very low solubility in any solvent, which limits efficiency.

Mechano-chemistry (MC) is a promising approach with which to conduct the reactions of solids under mechanical impact (usually at ambient temperature). The MC treatment of solids results in the transfer of energy to reactants (also known as mechanochemical activation [18]) and causes their transformation to reactive forms. MC procedures are widely used in the synthesis of drugs [19] and processing of pharmaceuticals [19]. A mechano-chemical reaction can be conducted in the presence of a grinding fluid, also known as liquid-assisted grinding, LAG [20].

Amongst the instrumental analysis of biopharmaceuticals, including those processed by grinding [19], spectroscopic methods are widely used, especially infrared (IR) spectroscopy. The attenuated total reflectance Fourier-transform infrared (ATR-FTIR) spectroscopy is suitable for the analysis of pharmaceutical powders [21], including those prepared by grinding [22].

It is of importance to study various physical forms of bonded drugs, such as pharmaceutical powders and shaped bodies (i.e., pellets). In most reports, the drug is released from a specimen in the physical form of powder, while shaped bodies are suitable for drug-eluting implants. An example of a pellet-shaped drug-eluting implant is a Gliadel wafer approved by the FDA to treat brain cancer [23].

Further, it is important that pharmaceutical shaped bodies are stable for a long time in the psychological solution. The pelletization of MOFs was reported for sorption and catalysis [24]. To our knowledge, there are no reports of pharmaceutical pellets containing any MOFs and drugs, which shows long-term stability in physiological buffers. To improve the stability of functional pressed pellets, a small amount of binder is used, such as carboxymethyl cellulose (CMC) and its salts [25]. However, there are no reports of using CMC to make pellets of MOFs for any application. Moreover, no studies have been published on the delayed release of anti-cancer drugs from MOFs in the physical shape of a pressed pellet.

Delayed drug release is studied in vitro to liquid drug release medium—the buffer solution, e.g., PBS [26], at 37 °C. Most often, manual periodic sampling is practiced, when a small aliquot of the suspension of the release medium is periodically collected from the dissolution vessel [27] for analysis. While this method is simple, it is prone to operator errors. Alternatively, an automated drug dissolution system (ADDS) can be utilized, which offers much better reproducibility and convenience. Namely, the ADDS automatically collects small aliquots from the drug release medium on a pre-defined schedule and temporarily stores them in an autosampler, ready for the subsequent off-line analysis by HPLC. The ADDS has been utilized in studies on the timed dissolution or release of pharmaceutical composites in the physical form of powders and pellets [28]. To our knowledge, ADDS has not been reported in studies on the stability of pellets containing the composites “Matrix/Drug” or the delayed release of anti-cancer drugs from pellets containing MOFs.

The response of leukemia cells to drugs, and specifically 6-TG, was tested on multiple cell lines including MOLT-4, CCRF-CEM, and Wilson [29], but the formation of colonies with MV-4-11 cells and their response to the treatment by 6-TG or its composites have not been reported.

Previously [30], we reported the stoichiometric encapsulation complexes of aluminum MOF Basolite A100 aka MIL-53(Al), including its complex with DMSO, prepared by sorption [31]. We also reported the transformation of tautomers of the anti-cancer drug gemcitabine during the LAG, using ATR-FTIR spectroscopy and XRD [32].

Here, the following data are reported. First, we present the interaction of 6-TG with the powder of Al-MOF DUT-4 by liquid-assisted grinding (LAG), with the formation of a new pharmaceutical composite. Second, we have a study of the obtained composite (6-TG)(DUT-4) by complementary ATR-FTIR spectroscopy and powder XRD. Third, we investigated the delayed release of 6-TG, with its temporal release profile and kinetics for this composite in the form of powder to PBS at 37 °C under stirring. Fourth, the preparation of a novel robust pharmaceutical pellet containing this composite is described, using organic binder carboxymethylcellulose (CMC), which has previously not been reported for this purpose. Fifth, we carried out testing the long-term stability of this pellet in PBS at 37 °C under stirring, using time-programmed automated in situ image collection. Sixth, the temporal release of the 6-TG from such a pellet is studied on a time scale of up to 10 days. Finally, the progressive and time-dependent suppression of the colony survival of MV-4-11 leukemia cells is presented by the composite versus pure 6-TG in a prolonged time scale of up to 9 days.

## 2. Materials and Methods

### 2.1. Chemicals

All chemicals in this experiment were of reagent grade or better. 2,6-naphthalenedicarboxylic acid was of ≥98% purity (TCI America, Portland, OR, USA, product N0377), N,N-dimethyl formamide was of 97% purity (VWR, Radnor, PA, USA, product TCD0722-500ML), and Al(NO_3_)_3_·9H_2_O was of 99+% purity (Fisher Scientific, Waltham, MA, USA, product AC218281000). 6-thioguanine (6-TG) was of ≥98% purity (ChemImpex, Wood Dale, IL, USA, product 01491). Isopropanol was of 99% reagent grade (from VWR, Radnor, PA, USA, product number 470301-474).

The materials for preparing the mobile phase in HPLC-UV were potassium dihydrogen phosphate of HPLC grade (Fisher Scientific, Waltham, MA, USA, product AC447370500) and methanol of HPLC grade (Fisher Scientific, product A452SK-4).

### 2.2. Synthesis and Activation of DUT-4 as Matrix

Synthesis of DUT-4 was conducted as published [33] and the as-synthesized material asisDUT-4 has been activated (to remove volatile impurities) at 190 °C for 4 h in a vacuum oven (Across International, Livingston, NJ, USA, model AT09e.110) connected to the two-stage vacuum oil pump (Xtractor Depot, pumping speed 12 cfm). Then, the activation oven has been vented with argon gas, and the vial with sample was promptly closed with a pre-weighted aluminum foil and plastic screw cap while still in the oven. Next, it was weighed to determine the loss of mass on activation and sealed with Parafilm tape to protect from moisture in ambient air. The activated sample is denoted actDUT-4. The 3D image of the structural unit of DUT-4 can be found in [33]. Figure S1 shows the simplified 2D image of the structural unit, with linker and metal cation.

### 2.3. Mechano-Chemical Interaction of 6-TG with DUT-4

In the first type of experiment, the interaction of 6-TG with a matrix of DUT-4 was performed by direct mixing of components in a glove bag (from NPS Corp, Green Bay, WI, USA, model Spilfyter, product 690341) filled with dried air. Namely, 1 mmol (167 mg) 6-TG was mixed with 1 mmol (258 mg) actDUT-4 and 0.25 mL isopropanol (grinding fluid) in a 5 mL stainless steel grinding vessel (Retsch), where one stainless steel grinding ball (7 mm in diameter) was added. The grinding in the mechanical grinding ball mill (model MM 301 Qiagen TissueLyser (Retsch GmbH & Co. KG, Haan, Germany) was conducted at 30 Hz by the repeating sequences “grind–idle” (5 min “grind”, followed by 5 min “idle”) until the total grinding time was 1 h.

In the second type of experiment, prior to LAG, an additional step of pre-mixing was conducted. Namely, working in a glove bag filled with dried air, 1 mmol (167 mg) 6-TG and 1 mmol (258 mg) actDUT-4 were added to a 5 mL stainless steel grinding vessel (Retsch). This corresponded to stoichiometric composite (6-TG)(DUT-4). The vessel was closed tightly with a stainless steel cap, and this assembly was placed in the 5 mL grinding vessel of the mechanical ball mill without a grinding ball, and shaking was performed at 30 Hz for 30 min.

After the pre-mixing step, the grinding vessel was opened in a glove bag under dried air, and, to the pre-mixed powders, 0.25 mL of isopropanol (grinding fluid) was added. Then, one stainless steel grinding ball (7 mm in diameter) was added, the grinding vessel was closed with a cap, and this assembly was ground at 30 Hertz. The grinding was conducted by the repeating sequences “grind–idle” (5 min “grind”, followed by 5 min “idle”) and this sequence was repeated until a total grinding time of 1 h. Then, the grinding vessel with the obtained wet mixture was opened and outgassed overnight to remove LAG fluid, in a vacuum desiccator connected to a diaphragm pump. The obtained composite was stored in a vacuum desiccator.

### 2.4. Instrumental Analysis of Powdered Specimens

ATR-FTIR spectra were collected by spectrometer Nicolet iS20 (Thermo Fisher Scientific, Madison, WI, USA) equipped with an ATR attachment model Smart iTX (from Thermo Fisher Scientific). For data collection, OMNIC software version 9.2.86 was used, the optical aperture was “Open”, and a variable Gain setting was used. Each IR spectrum was averaged 512 times, the spectral resolution was 2 cm^−1^ and the increment of the wavenumber was 0.24 cm^−1^. To minimize the adverse effects of humidity in the air, the interior of the FTIR spectrometer had been continuously purged with dried air at a flow rate of 30 scfh (standard cubic feet per hour); this was measured by a flowmeter model RMA-7 (Dwyer Instruments, LLC, Michigan City, IN, USA).

Dried air was prepared using an FT-IR Purge Gas Generator model 74-5041 (Parker Hannifin Corporation, Haverhill, MA, USA) with resultant water vapor content (per specifications) equivalent to a dewpoint of −73 °C (with relative humidity RH < 1%). To continuously monitor the quality of spectra and automatically remove artifacts due to traces of water vapor, the “Atmospheric Correction” parameter was enabled, and the “Spectral Quality Results” parameter was set at “H_2_O level” ≥95%. The ATR-FTIR spectra were presented in absorbance mode.

Powder X-ray diffraction (XRD) patterns were obtained using the diffractometer model MiniFlex (from Rigaku Corporation, Tokyo, Japan) which was equipped with a nickel foil filter to remove K-beta radiation. The copper K-alpha line at 0.15418 nm was used; the increments of the 2θ angle were 0.02 deg.

The numeric peak fitting of ATR-FTIR spectra was performed by Microcal Origin 2016 program.

### 2.5. Delayed Release of the Drug from Powders

Tests of delayed drug release were performed using an ADDS consisting of automatic dissolution tester (model VK 7000), heater/circulator (model VK 750D), peristaltic pump (model VK 810), and the automatic dissolution sampling station (model VK 8000), all from VanKel Industries, Edison, NJ, USA.

The procedure was similar to that in [34]: the paddle method was used with a stirring speed of 200 rpm. Here, the dissolution medium was 750 mL of 1X PBS without calcium and magnesium, prepared by dilution of PBS powder (Albert Bioscience Inc., Laguna Hills, CA, USA) with deionized water at a final pH of 7.4.

The dissolution medium in a one-liter glass dissolution vessel was maintained at 37 ± 0.5 °C. To avoid withdrawal of powder from suspension and blockage of the sampling station, the sampling cannulas of VK 7000 dissolution apparatus were equipped with 10 µM porous filters (made of UHMW polyethylene, product FIL010-01-a from Quality Lab Accessories).

The matrix actDUT-4 has formula C_10_H_6_(CO_2_^−^)_2_Al^3+^(OH^−^) and formal molar mass 214.2 + 27.0 + 17.0 = 258.2 mg/mmol, while 6-TG has a molar mass 167.2 mg/mmol. To obtain the equimolar amounts of compounds, the initial mixture corresponded to molar mass 258.2 + 167.2 = 425.4 mg/mmol and formal composition (6-TG)(DUT-4). Correspondingly, the content of 6-TG was 100% × 167.2/425.4 = 39.3 wt.%.

In drug release experiments from powder, the specimens contained ca. 33 mg of 6-TG (for pure drug) or the proportional amount of composite. Experiments were conducted under sink conditions [34] and molar concentration of 6-TG was always at least 3 times less than its solubility. The ADSS was turned on, the stirring of PBS was started in the dissolution vessel, and the powdered specimen was promptly dropped into it. Then, at pre-determined time intervals (45 min or 15 min at first, then 240 min at longer times), the 2 mL aliquots were automatically withdrawn and transferred to VK 8000 sampling station. The collected samples were frozen at −80 °C and stored until their batch analysis was conducted by HPLC-UV method.

### 2.6. Chromatographic Analysis of Released Thioguanine

The frozen samples were thawed and filtered by a PTFE syringe filter (0.22 μm pore size and 4 mm diameter) with Luer–Lok connectors (Tisch Scientific, Cleves, OH, USA, product SF17504) using disposable 1 mL Luer Lock Tip Syringes (BH Supplies, Jackson, NJ, USA). The concentration of 6-TG in the filtered samples was determined by HPLC-UV method, by an instrument of series 1100 (Agilent Technologies Inc., Santa Clara, CA, USA) and Chemstation software version B.04.02.

A reverse-phase HPLC column of model ZORBAX Eclipse XDB-C18 (from Agilent Technologies Inc., Santa Clara, CA, USA, product 993967-902, 4.6 × 150 mm, 5 μm) was equipped with a guard cartridge (Agilent Technologies Inc., product 820950-925). It was also equipped with a G1314A variable wavelength detector. The mobile phase was a 25:75 *v*/*v* mixture of methanol and a 1.36% solution of potassium dihydrogen phosphate, and the detection wavelength was 254 nm. The flow rate of the mobile phase was 0.5 mL/min and the injection volume was 1 µL. The calibration plot of 6-TG for the HPLC-UV analysis was prepared using standard solutions of 6-TG in PBS.

### 2.7. Preparation of Pressed Pellets

A commercial stainless steel pellet pressing die assembly 1⁄4 inch in diameter (model EQ-Die-06D-B from MTI Corp., Richmond, CA, USA) has been used (Figure S2).

The 2% wt. aqueous solution of non-toxic binder (sodium salt of carboxymethyl cellulose, from ACROS Organic, product 332621000) was used. It was prepared by sonication (sonicator model VGT-1620QTD, from GT Sonic, China) until full dissolution and stored frozen until needed. Immediately before preparation of the pellet, the solution of binder was thawed.

First, the pellet pressing die assembly (bottom die insert and body of pressing die, but without top-pressing die, Figure S2) was placed on the plate of semi-analytical lab balance with mass range of 0–500 g and accuracy of 0.001 g (Bonvoisin brand), and tared. Second, a 20 mg solution of binder was added on top of the bottom-pressing die inside an assembly. Third, a sample of 50 mg of powder of composite (6-TG)(DUT-4) was added. Fourth, a 20 mg solution of binder was added on top of the powder in the assembly; then, the top-pressing die with a few rubber O-rings was inserted and pressed firmly.

Next, the pellet pressing die assembly with sample was fixed in the 12-speed benchtop drill press (the 10-inch model, from Bilt Hard, City of Industry, CA, USA). In it, the top-pressing die was fixed and used as a drill bit; the content of the pellet pressing die assembly was homogenized for 5 min by spinning the top-pressing die at a revolution rate of 300 rpm (rounds per minute).

Then, a pressing assembly with a homogenized paste was placed between the plates of a benchtop hydraulic press (from Carver, Wabash, IN, USA) and a pressure of 3 tons was applied. After 2 h, the pressure was released, and the pressing assembly with its content was outgassed (to evaporate water) overnight in a vacuum desiccator equipped with a manometer and a 2-stage oil-free vacuum pump (maximum flow 50 L/min, best vacuum −85 kPa, model HZW-165, brand Win Outdoor). Finally, the obtained outgassed pellet was removed from the pressing assembly and stored in a closed jar, to protect it from ambient humidity.

### 2.8. Delayed Drug Release from Pellets

The procedure was similar to that for powders except for the following details. First, the typical pellet was ca. 40 mg, and, for the composite, it contained 13 mg of 6-TG. Second, to monitor the integrity of the pellet during tests, periodic image capture of the interior of the dissolution vessel was conducted, using a webcam (high resolution, 1080 pixels, full HD, model N5, from Ziqian, China) and Yawcam software version 0.7.0; every 30 min a digital image was obtained and automatically saved on a PC.

### 2.9. Protocol for Clonogenic Assay of MV-4-11 Cells

MV-4-11 cells expressing YFP-Luciferase were the kind gift of Dr. Sharyn Baker from the Ohio State University (OHSU), USA. Cells were maintained in RPMI supplemented with 10% fetal bovine serum (FBS) and 2 mM glutamine. First, the dilution of drugs using DMSO as the vehicle was performed; after this incubation period, cells were counted using a 1:2 dilution with trypan blue (10 µL of cells with 10 µL of trypan blue) and the Countess Automated Cell Counter (Thermo Fisher Scientific, Waltham, MA, USA). Based on the total cell count, the required number of cells to achieve 7500 cells per well for each treatment and control group was collected. Then, the cells were centrifuged at 300× *g* for 5 min and resuspended in 1 mL of media. For each treatment condition, a 1:1 mixture of 1.25 mL methylcellulose and 1.25 mL cell culture media was prepared in a 5 mL fluorescence-activated cell sorting (FACS) tube and vortexed vigorously. Subsequently, 0.5 mL of cell suspension was added to this mixture and mixed briefly. The resulting suspensions were then distributed into a 24-well plate at 500 µL per well, with four replicates for each condition. The plates were incubated with 6-TG or the composite (as suspensions in DMSO) for up to 17 days. The periodic readings were taken at various time intervals using Celigo Image Cytometer (Revvity Inc., Boston, MA, USA).

## 3. Results and Discussion

### 3.1. Characterization of the Composite vs. Pure 6-TG

The mechano-chemical interactions of the molecules proceed by a heterogeneous reaction “solid–solid”, i.e. between compounds in the physical shape of powder. Mixtures of powders have a high heterogeneity, and mixing can be highly non-uniform in the mechano-chemical process. It was noted in a recent paper [35] “the non-uniform time-dependent distribution of the sample throughout the milling jar” and “an extremely non-uniform sample distribution in the jar”. In this work, when the LAG was conducted without a pre-mixing step, the poor mixing of the powders of reactants was detected (Figure S3a); the DUT-4 matrix is yellow (in the specimen jar) while the 6-TG drug is white (on the plastic lid of the specimen jar, with a stainless steel grinding ball). When LAG was conducted with pre-mixing (Figure S3b), the obtained sample was of a uniformly colored appearance. Hence, the pre-mixing step was always conducted before LAG.

Figure S4 shows the survey ATR-FTIR spectrum of 6-TG as the reactant in the LAG and Figure S5 shows the most important ranges of the ATR-FTIR spectra of this compound. The IR peak maxima are consistent with the reported [36] FTIR spectra of 6-TG in the form of the amino-thione tautomer (see Figure 1) that is present in crystals of this compound.

Table S1 shows the observed and reported IR peaks of 6-TG and their assignment to specific functional groups [36]. The wavenumbers of certain IR peaks of 6-TG can be used to understand the mode of bonding 6-TG molecules to the DUT-4 matrix in the composite, via their characteristic peak shifts. But it is first necessary to learn about those characteristic IR peaks of DUT-4 as a matrix.

Figure S6 shows the survey ATR-FTIR spectrum of the activated DUT-4, which is consistent with the literature [33].

Figure S7 shows the ranges of the IR spectra of DUT-4 in the same wavenumber scale as for 6-TG (Figure S5).

In the IR spectra of DUT-4, the peak at 3068 cm^−1^ is due to the C-H stretch vibrations of the aromatic ring. The peak at 1582.7 cm^−1^ is due to the asymmetric vibration ν_asym_(COO) in the linker of Al–MOFs including the structurally similar MIL-53(Al) [37]. The peak at 1430 cm^−1^ is due to the symmetric vibration of the same group [38], and the peak at 987 cm^−1^ is due to the deformation vibration δ(μ-OH) of the free (Al)O-H group as reported [37] for MIL-53(Al).

**Figure 2:**
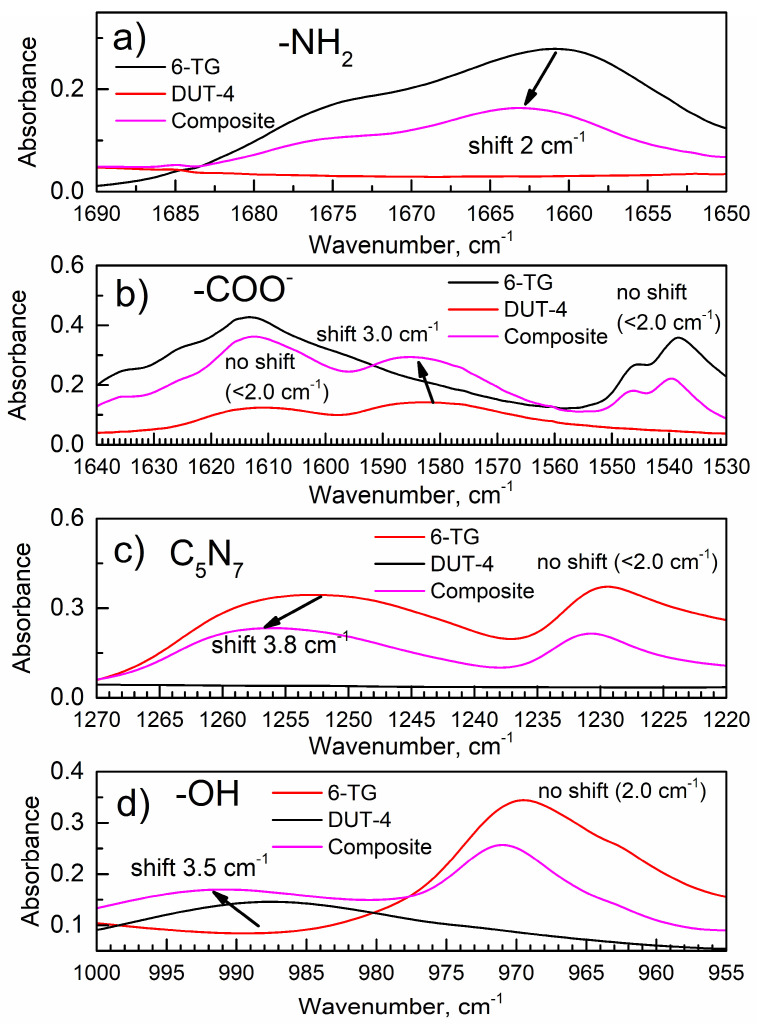
Select ranges of ATR-FTIR spectra of 6-TG, DUT-4, and composite with groups: (**a**) the NH_2_ group of 6-TG; (**b**) the COO^−^ group of DUT-4; (**c**) the C_5_H_7_ bond of 6-TG; and (**d**) the OH group of DUT-4.

Figure S8 shows the IR spectra of 6-TG, DUT-4, and the composite in the wavenumber ranges as in Figures S5 and S7, while Figure 2 shows the certain zoomed IR ranges. Here, significant peak shifts are observed after the formation of the composite by LAG, and their assignments are shown in Table S1.

Table 1 shows the IR peaks of functional groups in the 6-TG drug and DUT-4 matrix, which are affected by bonding and undergo shifts after the LAG. Here, only peak shifts in excess of 2 cm^−1^ (resolution of the IR spectrometer; see Section 2) are shown.

For the 6-TG drug, peaks due to vibration of the HNH (amino, NH_2_) group and bond between the C5 and N7 (C_5_N_7_) atoms in Figure 1 of the heterocyclic ring are shifted when the composite is formed; this indicates that these groups interact with the DUT-4 matrix. For the DUT-4 matrix, peaks due to vibrations of the COO^−^ and δ(μ-OH) group (see Figure S1) are shifted in the spectrum of the composite; hence, these groups interact with the 6-TG drug. Importantly, the IR peaks of only a few specific groups are shifted for the composite, as shown by the absence of shifts of other (not interacting) groups (see Figure 2). If these peak shifts were from the FTIR instrumental artifact, all peaks would have been shifted in the spectrum of the composite vs. the respective pure compounds, which is not the case.

At the same time, the similarity of many IR peaks of 6-TG in the pure form and composite form indicates that only a fraction of all 6-TG is bonded to DUT-4. The interaction of 6-TG and DUT-4 during the LAG can be described by the equation:

x 6-TG (s) + y DUT-4 (s) → (6-TG)_x_(DUT-4)_y_ (s) (1)

where (s) means solid state and (6-TG)_x_(DUT-4)_y_ denotes the complex. The latter is present in the composite together with non-bonded 6-TG and DUT-4.

**Figure 3:**
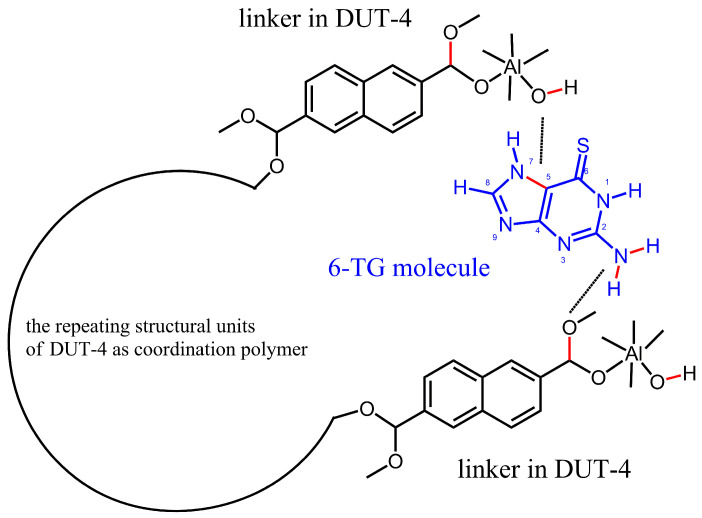
Proposed bonding of 6-TG drug to DUT-4 matrix.

Figure 3 shows the proposed bonding of the 6-TG molecule to DUT-4; for convenience, atoms are numbered as in Figure 1.

This model is consistent with a large orthorhombic lattice [33] of the DUT-4 matrix, with crystal lattice parameters a = 18.825(3) Å, b = 6.7867(5) Å, and c = 16.901(2) Å. The published data, namely, Figure 3 and Table 1 in Ref. [33], therefore, allow us to estimate the distance between the two linkers of DUT-4 via a simple calculation. This is a half-length of the diagonal of a rectangle with sides a = 18.825(3) Å and c = 16.901(2) Å, and, therefore, it is about 12.5 Å. This is about a half-size of the nanopore in DUT-4.

The distances involving the bonding of the 6-TG molecule to the DUT-4 matrix are roughly estimated as follows. First, the largest molecular dimension of the 6-TG molecule is ca. 7.5 Å as determined by the ChemDraw 3D program. Second, a strong hydrogen bond in Figure 3 between the NH_2_ group of 6-TG and the polar C-O bond in DUT-4 is estimated at 2.7 Å. Third, a polar non-covalent bond between the polar OH group of DUT-4 and the polar C-N bond in the 6-TG molecule is estimated at 2 Å. Therefore, the sum of the estimated distances is 7.5 Å (the molecule) + 2.7 Å (the first bond to the matrix) + 2 Å (the second bond to the matrix) = 12.2 Å. This is consistent with the above estimate of the distance between the two linkers in DUT-4 at about 12.5 Å, which is sufficient for the inclusion of the 6-TG molecule. Additionally, DUT-4 has a very large [33] Langmuir surface area of 1996 m^2^ g^−1^ and also a large pore volume of 0.68 cm^3^ g^−1^, which would favor the inclusion of 6-TG molecules into nanopores of DUT-4. This makes DUT-4 and similar MOFs attractive matrices for the inclusion of 6-TG and related small drug molecules, compared to “conventional” matrices such as alumina or silica.

**Figure 4:**
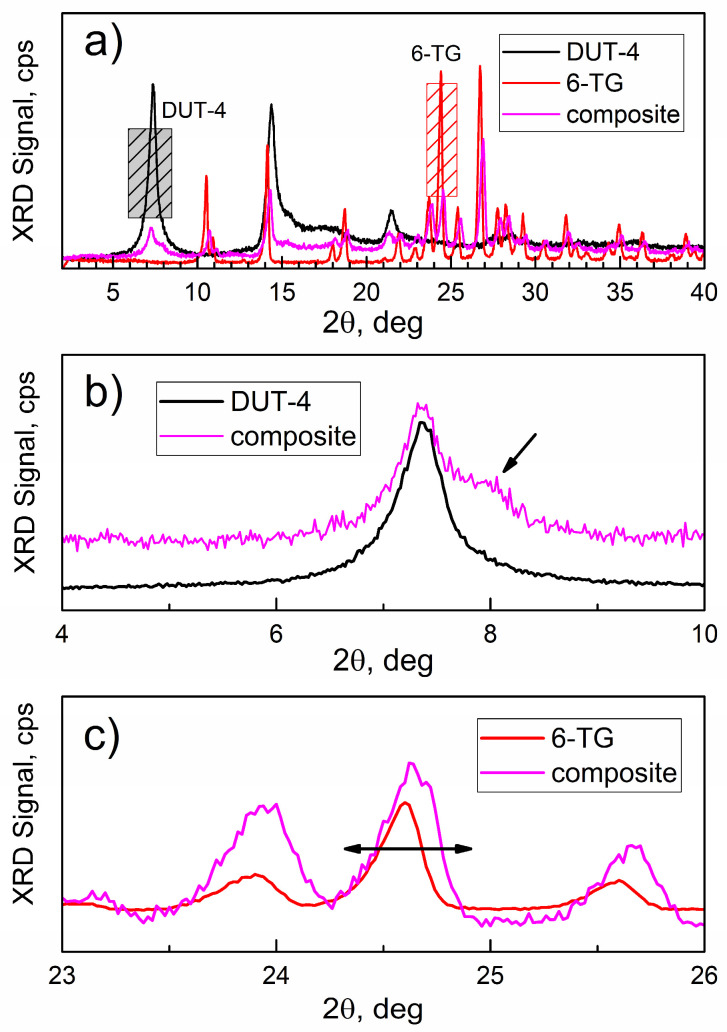
Powder XRD patterns: (**a**) the survey scans; (**b**) evolution of characteristic DUT-4 peak; and (**c**) evolution of characteristic 6-TG peak.

Figure 4 shows the powder XRD patterns of DUT-4, 6-TG, and the composite. In Figure 4a, the XRD pattern of DUT-4 is consistent with the literature [39], and its strongest and most characteristic peak is at ca. 7.4 deg. Importantly, there are no XRD peaks of 6-TG in the same range, so this peak is characteristic of DUT-4 (gray-shaded area). The pattern of 6-TG is also consistent with the literature [8]; the majority of its characteristic peaks (not overlapping with peaks of DUT-4) are at 2θ > 20 deg, consistently with a smaller molecular and crystal lattice size. The characteristic XRD peak of 6-TG at 24.5 deg is marked with the red shaded area.

Figure 4b shows a comparison of the characteristic peak of DUT-4 as the matrix, in pure DUT-4, and in the composite. For the XRD peak of the composite, a significant broadening is observed as well as a new shoulder at ca. 8 deg (marked with the arrow), due to the interaction with 6-TG molecules.

Figure 4c shows a comparison of the characteristic peak of 6-TG for the pure drug and the composite; broadening is observed (an arrow) that confirms the interaction between the two compounds.

Based on the FTIR and XRD data, one expects a quick dissolution of the “free” 6-TG drug present in the composite and the delayed release of 6-TG from the complex in the composite.

### 3.2. Delayed Release of 6-TG from Powder of Composite Versus Pure 6-TG to PBS

**Figure 5:**
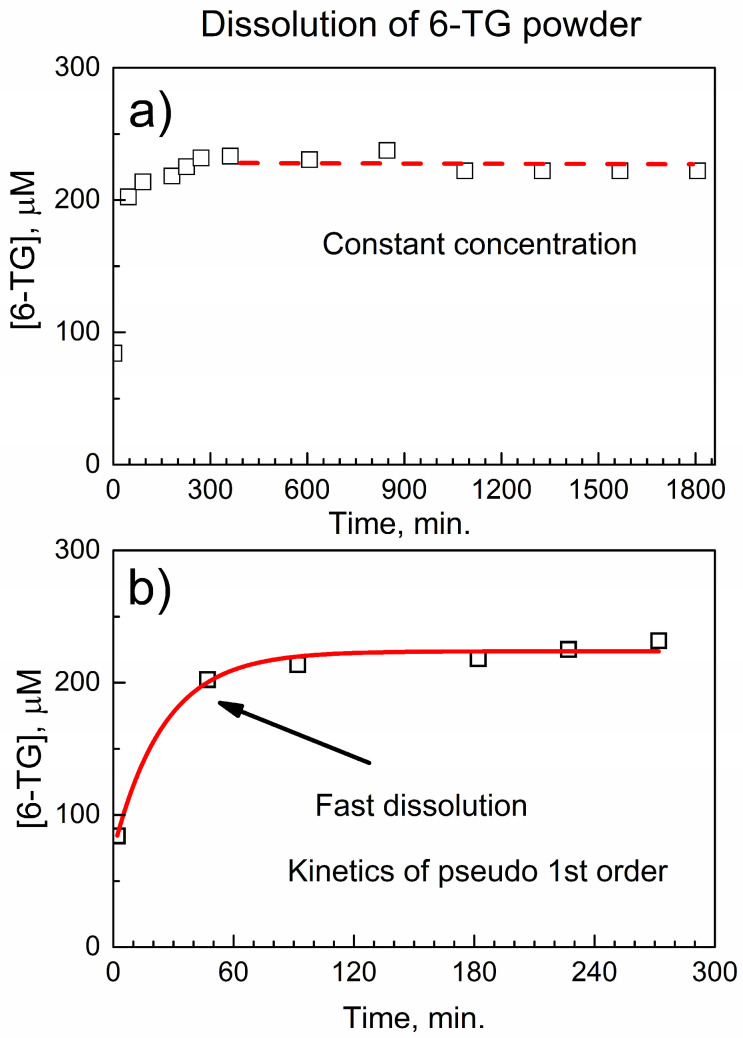
Temporal trace of dissolution of 6-TG powder in PBS at 37 °C: (**a**) drug dissolution curve; and (**b**) kinetic curve fitting of its initial stage.

Figure 5 shows the temporal trace of the dissolution of 6-TG powder (molar concentration in PBS at 37 °C versus time); this is the first control experiment. The concentration of 6-TG in PBS at each time point was determined from the HPLC-UV calibration plot of 6-TG, as can be seen in Figure S9.

In Figure 5a, the dissolution of 6-TG occurs relatively quickly within <300 min as expected for the pure drug, and then its concentration remains constant.

In Figure 5b, the drug release curve at the initial period (within ca. 300 min) has been fitted with the pseudo-first rate law [40]. The molar concentration [6-TG] of the dissolved drug corresponds to the product of reaction (2):

6-TG (s) → 6-TG (aq)(2)

where 6-TG (aq) indicates the 6-TG drug in PBS, with the kinetic formula y(t) = A + B × (1 − exp(−*k* × t)). Here, *k* is an effective kinetic rate constant of dissolution of the pure drug, B is the numeric coefficient, and A is an offset.

The kinetic curve in Figure 5b is well-fitted with an effective kinetic rate constant *k*_eff_(6-TG) = 0.040 ± 0.007 min^−1^ with a very good value of the adjusted goodness-of-fit parameter R^2^_adj_ = 0.985. The mechanism is likely the diffusion-limited dissolution of 6-TG under stirring.

Figure S10 shows data from the second control experiment, where the kinetics of dissolution of 6-TG powder was obtained after this drug had been processed by the LAG. This yields similar kinetics of dissolution as those for pure 6-TG, with the pseudo-first rate constant *k*_eff_(6-TG-LAG) = 0.032 ± 0.006 min^−1^ and a reasonable R^2^_adj_ = 0.964. Within the error, pure 6-TG and 6-TG after LAG give the same dissolution rates, which is consistent with the stability of 6-TG during the LAG.

**Figure 6:**
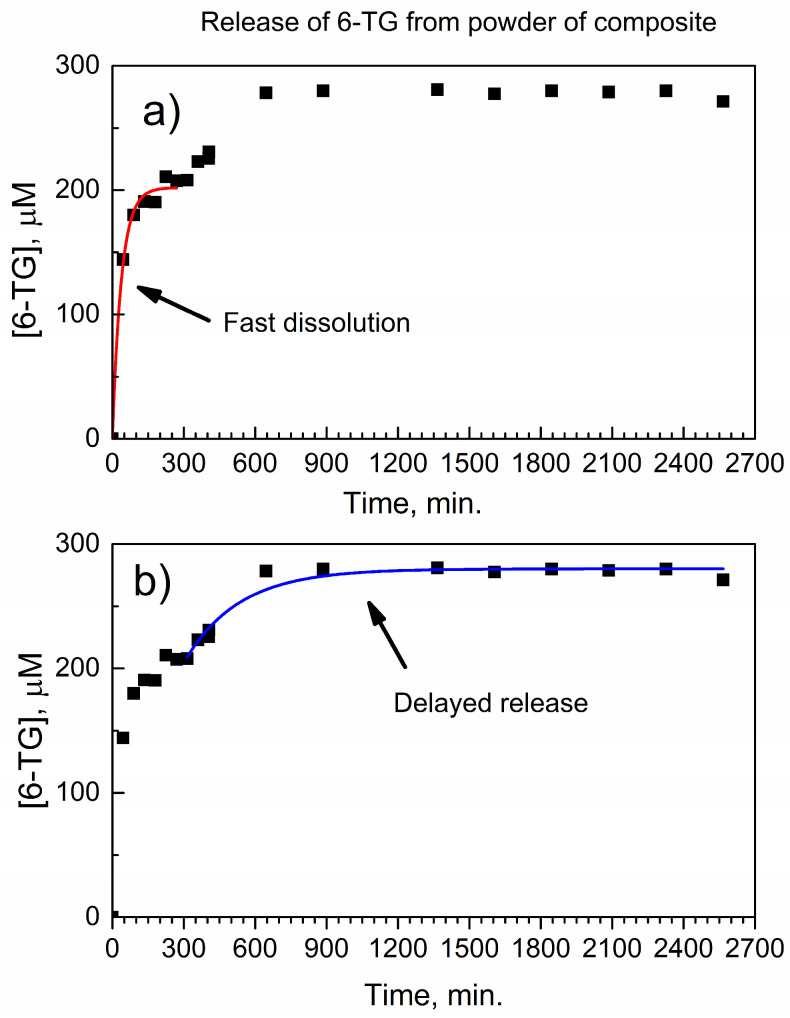
Temporal trace of release of 6-TG from composite with DUT-4 matrix in PBS at 37 °C: (**a**) with kinetic curve fitting of its early stage; and (**b**) with kinetic curve fitting of its late stage.

Figure 6 shows a temporal trace of the release of 6-TG from the composite with DUT-4; here, the specimen of the composite contains the same mass of 6-TG as the active ingredient, as in the control experiments above. The molar concentration [6-TG] when released from the composite increases on a longer time scale, compared to the control experiments in Figure 5 and Figure S10. More importantly, two distinct regimes are observed.

First, in Figure 6a within ca. 0–300 min, there is a trend similar to that of the pure drug. The fitting of the release curve results in the pseudo-first rate constant *k*_eff_(6-TGCompEarly) = 0.026 ± 0.003 min^−1^ and a very good R^2^_adj_ = 0.99. Within error, this is the same rate constant as observed for the dissolution of 6-TG after LAG (Figure S10) and it corresponds to the dissolution of a fraction of the total amount of 6-TG in the composite that is not bonded to the DUT-4 matrix (“free” drug).

Second, Figure 6b shows the delayed release at a longer time scale, which is fitted with a much smaller pseudo-first rate constant *k*_eff_(6-TGCompLate) = 0.0044 ± 0.0002 min^−1^ and R^2^_adj_ = 0.95. It is consistent with the delayed release of 6-TG bonded to the DUT-4 matrix in the composite; indeed, the rate constant *k*_eff_(6-TGCompLate) for the bonded drug is factor six times smaller (longer release) than *k*_eff_(6-TGCompEarly) for the non-bonded drug.

The composite contains both the non-bonded and bonded 6-TG drug. One can speculate, based on their relative amounts, whether the sorption takes place in the nanopores. Namely, Figure 6a shows that the total concentration of 6-TG in solution at the equilibrium is 280 micromole/L, while the non-bonded drug accounts for ca. 210 micromole/L. The difference of 70 micromole/L is due to the bonded drug or as much as 100% × 70/280 = 25% of 6-TG is bonded to DUT-4. This would correspond to the tentative formula of the complex (6-TG)_0.25_(DUT-4)_1_ or (6-TG)_1_(DUT-4)_4_. Given a large molar amount of the bonded drug, it is not likely that all the bonded drug is on the surface of DUT-4 and at least a fraction of it could be within the nanopores.

### 3.3. Delayed Release of 6-TG to PBS from the Pellet at Longer Time Scale

We showed above that the powder form of the composite features a time-delayed release of the 6-TG drug. For the potential use of these results in designing the prototype drug-eluting implant, it is important that the specimen is mechanically robust and does not disintegrate in the physiological liquid media. To our knowledge, there are no reports of pharmaceutical pellets containing any MOFs or any pharmaceutical drugs that show long-term stability in physiological buffers.

First, we assessed the temporal stability of a pressed pellet made of the composite (6-TG)(DUT-4) in PBS as the model physiological solution at 37 °C, under intense mechanical stirring for prolonged periods.

Figure S11 shows the photographic images of drug-releasing pellets at certain periods. In Figure S11a (top), the pellet is shown on the bottom of the dissolution vessel 60 min after the start of the test, and, in Figure S11b (bottom), it is shown at 4 days. The presented drug-eluting pellet maintains its shape well after being stirred in PBS.

**Figure 7:**
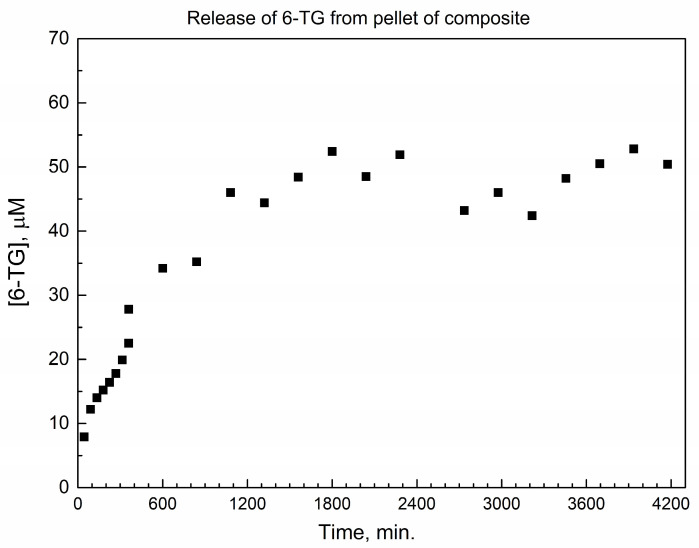
The early period of temporal trace of delayed 6-TG release from the pellet of composite to PBS at 37 °C (up to 2.9 days).

Figure 7 shows the temporal trace of the delayed drug release (molar concentration of 6-TG released to PBS at 37 °C versus time) from the pellet of composite. The molar concentration [6-TG] steadily increases from zero to ca. 2400 min, and then remains relatively constant; the dependence is complex and cannot be fitted with any simple rate law.

Importantly, the plateau of [6-TG] in Figure 7 is close to the therapeutically active concentration range of 6-TG in the plasma of patients receiving 1800 mg 6-TG as a 60 min infusion [41], namely, ca. 50 nmol/mL (μM) at infusion time and 1 h afterward. However, in ref. [41], the concentration of 6-TG sharply falls below 30 μM in less than 5 h (1800 min), while, in Figure 7, it persists for up to 2.9 days.

Figure S12 shows the representative temporal trace of delayed drug release from the pellet of composite at a longer time. To our knowledge, this is the longest time scale of delayed drug release (up to 10 days) which was reported by using an ADDS. In ref. [41], the concentration of 6-TG sharply falls to as low as ca. 3 μM within 30 h (1800 min), significantly below the therapeutically active concentration window. In contrast, in Figure S12, the pellet maintains a molar concentration [6-TG] within the 20–30 μM window for as long as 14,000 min (233 h) or an order of magnitude longer. This is despite the forced agitation in the drug dissolution vessel, which is not the case for the drug in the bloodstream. Therefore, this finding constitutes an advantage of the sustained 6-TG release by the presented drug-eluting pellet. In Figure S12, some decrease in [6-TG] over a very long period is likely due to the gradual hydrolysis and/or oxidation during agitation in the release vessel. Importantly, despite the significant amount of 6-TG free powder present in the pellet of the composite (6-TG)(DUT-4) in this work, the dissolution of this non-bonded drug does not impact the shape and mechanical stability of the drug-releasing pellet, as can be seen in Figure S11.

In terms of the applicability of the presented data to designing drug-eluting implants, the time scale for the delayed release of 6-TG from the pellet in Figure S12 within 0–10 days significantly exceeds the reported time scale for the delayed release of carmustine (anti-cancer drug) from Gliadel wafer. Namely, the delayed release of carmustine from Gliadel wafer in vivo to rat brain [42] occurs within 0–120 h (0–5 days). Hence, the described robust drug-eluting pellet could be used to deliver the therapeutically relevant concentrations of the 6-thioguanine drug to a patient, for the potential long-term treatment of acute myeloid leukemia [1] or Crohn’s disease [5].

### 3.4. The In Vitro Clonogenic Assay with MV-4-11 Leukemia Cells

**Figure 8:**
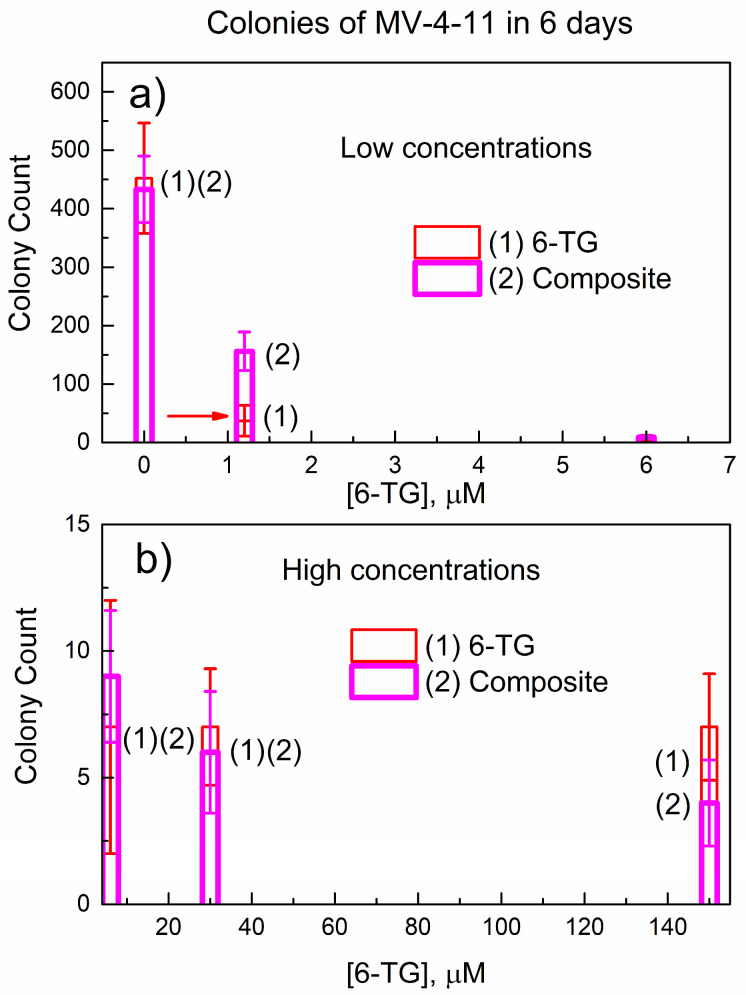
Colony count of MV-4-11 cells in 6 days after treatment with 6-TG or the composite: (**a**) lower concentration range; and (**b**) higher concentration range.

Figure 8 shows the data of the colony formation of MV-4-11 cells (colony count) at 6 days obtained by Celigo Image Cytometer (see Section 2); in 2 days after the start of the experiment, the number of colonies was too small.

As expected, for the control in Figure 8a, the colony count is, within error bars, the same for the treatment with 6-TG and the composite. At a low concentration of 1.2 μM, pure 6-TG (red) suppresses colonies more strongly than the composite (pink); this is indicated with a red arrow (this color denotes 6-TG).

Figures S13 and S14 show the representative images of the groups of MV-4-11 colonies treated with 6-TG and the composite. In Figure 8b, the intermediate concentration of 6 μM and higher concentrations (30 μM and 150 μM) give the same colony count, within error bars. The data for the intermediate concentration are shown, for convenience, in Figure 8a,b. Hence, at the early period of 6 days, the composite has no advantage vs. pure 6-TG in suppressing colony formation.

**Figure 9:**
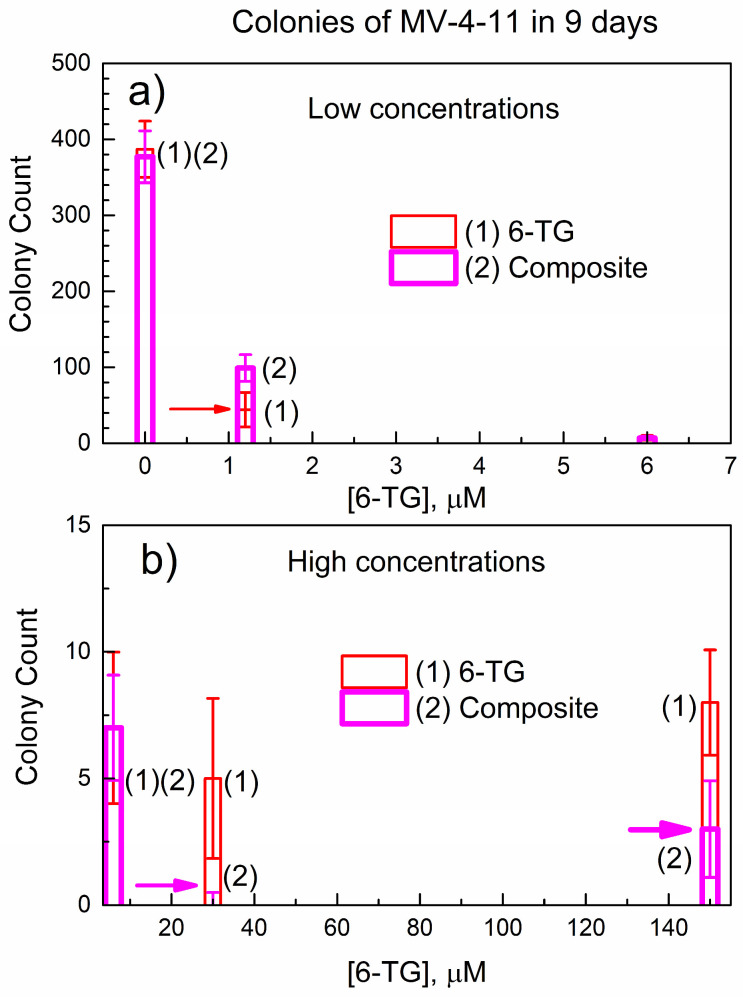
Colony count of MV-4-11 cells in 9 days after treating with 6-TG or the composite: (**a**) lower concentration range; and (**b**) higher concentration range.

Figure 9 shows the colony count of MV-4-11 cells at a longer exposure time of 9 days. In Figure 9a, the control gives the same colony count, and the lowest concentration of 1.2 μM results in stronger colony suppression versus the composite. The intermediate concentration of 6 μM (in Figure 9a,b) shows the same colony count for 6-TG and the composite.

However, for higher concentrations (30 μM and 150 μM), the trend has changed. Namely, the composite suppresses colony formation stronger than 6-TG; this is indicated by a horizontal pink arrow.

To our knowledge, there are no publications on the suppression of MV-4-11 colonies by pure 6-TG or its formulations. However, a study on the cytotoxicity of 6-TG against three related human leukemic cell lines (NOLT-4, CCRF-CEM, and Wilson) reports [29] the median IC50 = 20 μM in the time scale of up to 72 h. This reported concentration of 6-TG is consistent with the concentration range (30 and 150 μM) in Figure 9, where the change in the trend occurs.

A comparison of the data in Figure 8b and Figure 9b allows the conclusion that, at a longer exposure time of 9 days, the composite is more effective in suppressing colony formation than pure 6-TG. This finding is consistent with data on the in vitro delayed release of 6-TG from the powder of the composite to PBS, as can be seen in Figure 6. We performed additional colony counts at the exposure time of 17 days, and they are similar to Figure 9. The explanation of the change in the relative efficiency of colony suppression by 6-TG and the composite is as follows: the composite contains both free (non-bonded) 6-TG and 6-TG bonded in the complex (6-TG)_x_(DUT-4)_y_. At lower concentrations of the composite (1.2 μM and 6 μM), a free 6-TG in it is mainly responsible for colony suppression. However, at higher concentrations (30 μM and 150 μM) and a longer exposure time, the composite is more effective due to the delayed release of 6-TG.

## 4. Conclusions

The mechano-chemical interaction of 6-TG with the DUT-4 matrix by LAG results in the composite (6-TG)(DUT-4) in the form of powder. In the composite, molecules of 6-TG are bonded by the amino groups and C and N atoms of the pyrimidine ring to carboxylate and (Al)O-H groups in DUT-4, forming the complex. Moreover, free 6-TG and non-bonded DUT-4 are present in the composite. For pure 6-TG, the in vitro timed release to PBS at 37 °C in the automated drug dissolution system shows a relatively quick (<300 min) and complete dissolution, with the pseudo first-order rate law and constant *k*_eff_(6-TG) = 0.040 ± 0.007 min^−1^. In contrast, the composite shows a quick dissolution of non-bonded 6-TG with the first-order rate constant *k*_eff_(6-TGCompEarly) = 0.026 ± 0.003 min^−1^, followed by a much slower release of the bonded drug, with the first-order rate constant *k*_eff_(6-TGCompLate) = 0.0044 ± 0.0002 min^−1^. The pharmaceutical pressed pellet prepared from the composite shows good mechanical stability in the in vitro timed release for up to 10 days, and the sustained release of 6-TG at the therapeutically usable window of 20–30 μM. The periodic count of colonies of leukemia MV-4-11 cells by the Celigo Image Cytometer for up to 9 days shows the higher efficiency of the composite versus pure 6-TG in suppressing the proliferation of colonies. Aluminum MOFs are promising matrices for 6-thioguanine and other small-molecule antimetabolites for a controlled delayed release, and they show potential for the preparation of drug-eluting implants.

## Figures and Tables

**Table 1:** The IR wavenumbers of peaks of 6-TG, DUT-4, and composite that undergo changes.

**6-TG. Wavenumber, cm^−1^**	**IR Mode of 6-TG (Figure 1)**	**Composite. Wavenumber; Shift, cm^−1^**	**DUT-4. Wavenumber, cm^−1^**	**Assignment of DUT-4 Peak (Figure S1)**
1661.0	HNH (NH_2_)	1663.5; Shift vs. 6-TG: 1663.5 − 1661.0 = +2.5	-	-
	-	1585.4; Shift vs. DUT-4: 1585.4 − 1582.7 = +2.7	1582.7	ν_asym_(COO^−^)
1252.3	C_5_N_7_	1256.1; Shift vs. 6-TG: 1256.1 − 1252.3 = +3.8	-	-
-	-	991.0; Shift vs. DUT-4: 991.0 − 987.5 = +3.5	987.5	δ(μ-OH)

**6-TG. Wavenumber, cm^−1^**

**IR Mode of 6-TG (Figure 1)**

**Composite. Wavenumber; Shift, cm^−1^**

**DUT-4. Wavenumber, cm^−1^**

**Assignment of DUT-4 Peak (Figure S1)**

## Data Availability

Data are contained within the article and Supplementary Materials.

## Supplementary Materials

The following supporting information can be downloaded at: https://www.mdpi.com/article/10.3390/nano14191571/s1, Figure S1: The simplified molecular formula of the unit lattice of DUT-4 (encapsulation matrix); Figure S2: Commercial pellet pressing die and anvil for preparation of pressed pellets. Left: bottom die insert; center: body of pressing die; right: top-pressing die with few rubber O-rings; Figure S3: Photo of the mixture of reactants after LAG: (a) left: without pre-mixing step; and (b) right: with pre-mixing step; Figure S4: Survey ATR-FTIR spectrum of 6-TG; Figure S5: The ATR-FTIR spectra of 6-TG: (a) the high wavenumbers range; (b) the mid-IR range; and (c) the low wavenumbers range; Table S1: The wavenumbers of IR peaks of 6-TG with peak assignments; Figure S6: The survey ATR-FTIR spectrum of activated DUT-4 as matrix; Figure S7: The ATR-FTIR spectra of DUT-4: (a) the high wavenumbers range; (b) the mid-IR range; and (c) the low wavenumbers range; Figure S8: The ATR-FTIR spectra of 6-TG, DUT-4, and composite: (a) the high wavenumbers range; (b) the mid-IR range; and (c) the low wavenumbers range; Figure S9: The HPLC-UV calibration plot of 6-TG in PBS; Figure S10: Temporal trace of dissolution of 6-TG powder after LAG in PBS at 37 °C: (a) drug dissolution curve; and (b) kinetic curve fitting of its initial stage; Figure S11: Photos of drug-releasing pellet in the dissolution apparatus: (a) (top) in 60 min (image # 2); and (b) (bottom) in 5820 min or 4 days (image # 194); Figure S12: Extended temporal trace of delayed release of 6-TG from pellet of composite to PBS at 37 °C up to 10 days; Figure S13: Representative images of the treatment groups of MV-4-11 cell colonies with 6-TG: left: control; middle: 1.2 μM; and right: 6.0 μM; Figure S14: Representative images of the treatment groups of MV-4-11 cell colonies with composite: left: control; middle: 1.2 μM; and right: 6.0 μM.

## References

[1] Kohlschütter J., Michelfelder S., Trepel M. (2008). Drug delivery in acute myeloid leukemia. Expert Opin. Drug Deliv..

[2] Stork L.C., Matloub Y., Broxson E., La M., Yanofsky R., Sather H., Hutchinson R., Heerema N.A., Sorrell A.D., Masterson M. (2010). Oral 6-mercaptopurine versus oral 6-thioguanine and veno-occlusive disease in children with standard-risk acute lymphoblastic leukemia: Report of the children’s oncology group CCG-1952 clinical trial. Blood.

[3] Rubin J., Schutt A.J., Pitot H.C.I. (1992). A phase II study of intravenous 6-thioguanine (NSC-752) in advanced colorectal carcinoma. Am. J. Clin. Oncol..

[4] Bayoumy A.B., Crouwel F., Chanda N., Florin T.H.J., Buiter H.J.C., Mulder C.J.J., de Boer N.K.H. (2021). Advances in thiopurine drug delivery: The current state-of-the-art. Eur. J. Drug Metab. Pharmacokinet..

[5] Meijer B., Mulder C.J., Peters G.J., van Bodegraven A.A., de Boer N.K. (2016). Efficacy of thioguanine treatment in inflammatory bowel disease: A systematic review. World J. Gastroenterol..

[6] Bhattacharjee S. (2021). Understanding the burst release phenomenon: Toward designing effective nanoparticulate drug-delivery systems. Ther. Deliv..

[7] Hsieh H.-C., Hung S.-C., Huang S.-Y., Huang F.-L., Chou C.-M. (2022). A prospective, randomized study assessing different modalities for flushing totally implanted vascular access device in children with malignancy. J. Chin. Med. Assoc..

[8] Sierpe R., Noyong M., Simon U., Aguayo D., Huerta J., Kogan M.J., Yutronic N. (2017). Construction of 6-thioguanine and 6-mercaptopurine carriers based on betacyclodextrins and gold nanoparticles. Carbohydr. Polym..

[9] Samokhvalov A. (2017). Adsorption on Mesoporous Metal-Organic Frameworks in Solution for Clean Energy, Environment and Healthcare.

[10] Horcajada P., Chalati T., Serre C., Gillet B., Sebrie C., Baati T., Eubank J.F., Heurtaux D., Clayette P., Kreuz C. (2010). Porous metal-organic-framework nanoscale carriers as a potential platform for drug delivery and imaging. Nat. Mater..

[11] Anand R., Borghi F., Manoli F., Manet I., Agostoni V., Reschiglian P., Gref R., Monti S. (2014). Host-guest interactions in Fe(III)-trimesate MOF nanoparticles loaded with doxorubicin. J. Phys. Chem. B.

[12] Lucena F.R.S., de Araújo L.C.C., Rodrigues M.D.D., da Silva T.G., Pereira V.R.A., Militão G.C.G., Fontes D.A.F., Rolim-Neto P.J., da Silva F.F., Nascimento S.C. (2013). Induction of cancer cell death by apoptosis and slow release of 5-fluoracil from metal-organic frameworks Cu-BTC. Biomed. Pharmacother..

[13] Samokhvalov A. (2018). Aluminum metal–organic frameworks for sorption in solution: A review. Coord. Chem. Rev..

[14] Grall R., Hidalgo T., Delic J., Garcia-Marquez A., Chevillard S., Horcajada P. (2015). In vitro biocompatibility of mesoporous metal (III; Fe, Al, Cr) trimesate MOF nanocarriers. J. Mater. Chem. B.

[15] Wolinsky J.B., Colson Y.L., Grinstaff M.W. (2012). Local drug delivery strategies for cancer treatment: Gels, nanoparticles, polymeric films, rods, and wafers. J. Control. Release.

[16] Nakahama M., Reboul J., Yoshida K., Furukawa S., Kitagawa S. (2015). L-glutamic acid release from a series of aluminum-based isoreticular porous coordination polymers. J. Mater. Chem. B.

[17] Dong H., He Y., Fan C., Zhu Z., Zhang C., Liu X., Qian K., Tang T. (2022). Encapsulation of imazalil in HKUST-1 with versatile antimicrobial activity. Nanomaterials.

[18] Uzunova-Bujnova M., Dimitrov D., Radev D., Bojinova A., Todorovsky D. (2008). Effect of the mechanoactivation on the structure, sorption and photocatalytic properties of titanium dioxide. Mater. Chem. Phys..

[19] Sović I., Lukin S., Meštrović E., Halasz I., Porcheddu A., Delogu F., Ricci P.C., Caron F., Perilli T., Dogan A. (2020). Mechanochemical preparation of active pharmaceutical ingredients monitored by in situ Raman spectroscopy. ACS Omega.

[20] Denlinger K.L., Ortiz-Trankina L., Carr P., Benson K., Waddell D.C., Mack J. (2018). Liquid-assisted grinding and ion pairing regulates percentage conversion and diastereoselectivity of the Wittig reaction under mechanochemical conditions. Beilstein J. Org. Chem..

[21] Tiernan H., Byrne B., Kazarian S.G. (2020). ATR-FTIR spectroscopy and spectroscopic imaging for the analysis of biopharmaceuticals. Spectrochim. Acta A.

[22] Grisedale L.C., Jamieson M.J., Belton P., Barker S.A., Craig D.Q.M. (2011). Characterization and quantification of amorphous material in milled and spray-dried salbutamol sulfate: A comparison of thermal, spectroscopic, and water vapor sorption approaches. J. Pharm. Sci..

[23] Perry J., Chambers A., Spithoff K., Laperriere N. (2007). Gliadel wafers in the treatment of malignant glioma: A systematic review. Curr. Oncol..

[24] Tsalaporta E., MacElroy J.M.D. (2020). A comparative study of the physical and chemical properties of pelletized HKUST-1, ZIF-8, ZIF-67 and UIO-66 powders. Heliyon.

[25] Van der Merwe M.C.J., Garbers-Craig A.M. (2017). Influence of a carboxymethyl cellulose (CMC) binder on the mechanical properties of iron ore pellets. J. South. Afr. Inst. Min. Metall..

[26] Du P.-Y., Gu W., Liu X. (2016). A three-dimensional Nd(III)-based metal-organic framework as a smart drug carrier. New J. Chem..

[27] Zhang F.-M., Dong H., Zhang X., Sun X.-J., Liu M., Yang D.-D., Liu X., Wei J.-Z. (2017). Postsynthetic modification of ZIF-90 for potential targeted codelivery of two anticancer drugs. ACS Appl. Mater. Interfaces.

[28] Rongthong T., Pongjanyakul T. (2021). Quaternary polymethacrylate−magnesium aluminum silicate film formers: Stability studies for tablet coatings. J. Drug Deliv. Sci. Technol..

[29] Adamson P.C., Poplack D.G., Balis F.M. (1994). The cytotoxicity of thioguanine vs mercaptopurine in acute lymphoblastic leukemia. Leuk. Res..

[30] Dai J., McKee M.L., Samokhvalov A. (2015). Fluorescence of A100 MOF and adsorption of water, indole, and naphthalene on A100 by the spectroscopic, kinetic, and DFT studies. J. Phys. Chem. C.

[31] Grinnell C., Samokhvalov A. (2019). The solid-state synchronous vs. Conventional fluorescence spectroscopy and complementary methods to study the interactions of aluminum metal-organic framework Basolite A100 with dimethyl sulfoxide. J. Lumin..

[32] Henry B., Samokhvalov A. (2023). Characterization of tautomeric forms of anti-cancer drug gemcitabine and their interconversion upon mechano-chemical treatment, using ATR-FTIR spectroscopy and complementary methods. J. Pharm. Biomed. Anal..

[33] Senkovska I., Hoffmann F., Fröba M., Getzschmann J., Böhlmann W., Kaskel S. (2009). New highly porous aluminium based metal-organic frameworks: Al(OH)(NDC) (NDC = 2,6-naphthalene dicarboxylate) and Al(OH)(BPDC) (BPDC = 4,4′-biphenyl dicarboxylate). Microporous Mesoporous Mater..

[34] Sievens-Figueroa L., Pandya N., Bhakay A., Keyvan G., Michniak-Kohn B., Bilgili E., Davé R.N. (2012). Using USP I and USP IV for discriminating dissolution rates of nano- and microparticle-loaded pharmaceutical strip-films. AAPS PharmSciTech.

[35] Halasz I., Friščić T., Kimber S.A.J., Užarević K., Puškarić A., Mottillo C., Julien P., Štrukil V., Honkimäki V., Dinnebier R.E. (2014). Quantitative in situ and real-time monitoring of mechanochemical reactions. Faraday Discuss..

[36] Kasende O.E. (2002). Infrared spectra of 6-thioguanine tautomers. An experimental and theoretical approach. Spectrochim. Acta A.

[37] Salazar J.M., Weber G., Simon J.M., Bezverkhyy I., Bellat J.P. (2015). Characterization of adsorbed water in MIL-53(Al) by FTIR spectroscopy and ab-initio calculations. J. Chem. Phys..

[38] Niekiel F., Ackermann M., Guerrier P., Rothkirch A., Stock N. (2013). Aluminum-1,4-cyclohexanedicarboxylates: High-throughput and temperature-dependent in situ EDXRD studies. Inorg. Chem..

[39] Krylov A.S., Shipilovskikh S.A., Krylova S.N., Slyusarenko N.V., Timofeeva M., Kenzhebayeva Y.A., Bachinin S.V., Yushina I.D., Cherepakhin A.V., Shestakov N.P. (2024). Application of DUT-4 MOF structure switching for optical and electrical humidity sensing. Dalton Trans..

[40] Simonin J.-P. (2016). On the comparison of pseudo-first order and pseudo-second order rate laws in the modeling of adsorption kinetics. Chem. Eng. J..

[41] Andrews P.A., Egorin M.J., May M.E., Bachur N.R. (1982). Reversed-phase high-performance liquid chromatography analysis of 6-thioguanine applicable to pharmacologic studies in humans. J. Chromatogr. B Biomed. Appl..

[42] Fleming A.B., Saltzman W.M. (2002). Pharmacokinetics of the carmustine implant. Clin. Pharmacokinet..

