# Supplementary material for: Interaction of 6-Thioguanine with Aluminum Metal–Organic Framework Assisted by Mechano-Chemistry, In Vitro Delayed Drug Release, and Time-Dependent Toxicity to Leukemia Cells
Source: Nanomaterials (Basel). 2024 Sep 29;14(19):1571. doi: 10.3390/nano14191571 (PMC11477990; doi:10.3390/nano14191571)
Supplement: Supplementary file 1 [file nanomaterials-14-01571-s001.zip › nanomaterials-3159066-supplementary.pdf]

Supplementary Materials

Interaction of 6-Thioguanine with Aluminum Metal–Organic Framework Assisted by Mechano-Chemistry, In Vitro Delayed Drug Release, and Time-Dependent Toxicity to Leukemia Cells

Sheriff Umar [1], Xavier Welch [1], Chihurumanya Obichere [1], Brandon Carter-Cooper [2] and Alexander Samokhvalov [1,*]

[1] Department of Chemistry, Morgan State University, 1700 East Cold Spring Lane, Baltimore, MD 21251, USA
[2] Translational Laboratory Shared Services (TLSS), The University of Maryland School of Medicine's & Greenebaum Comprehensive Cancer Center, 22 S. Greene Street, Baltimore, MD 21201, USA
Correspondence: alexandr.samokhvalov@morgan.edu; Tel.: +1-443-885-4963

1. Introduction

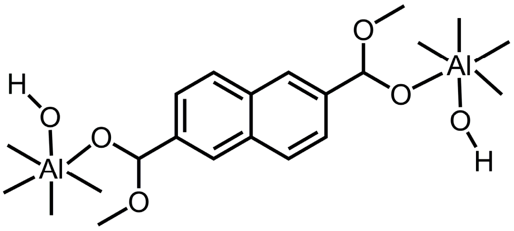

Figure S1. The simplified molecular formula of the unit lattice of DUT-4 (encapsulation matrix).

2. Materials and Methods

2.7. Preparation of Pressed Pellets

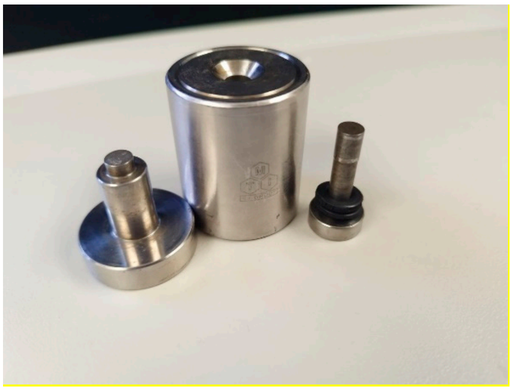

Figure S2. Commercial pellet pressing die and anvil for preparation of pressed pellets. Left: bottom die insert; center: body of pressing die; right: top-pressing die with few rubber O-rings.

3. Results and Discussion

3.1. Characterization of the Composite vs. Pure 6-TG

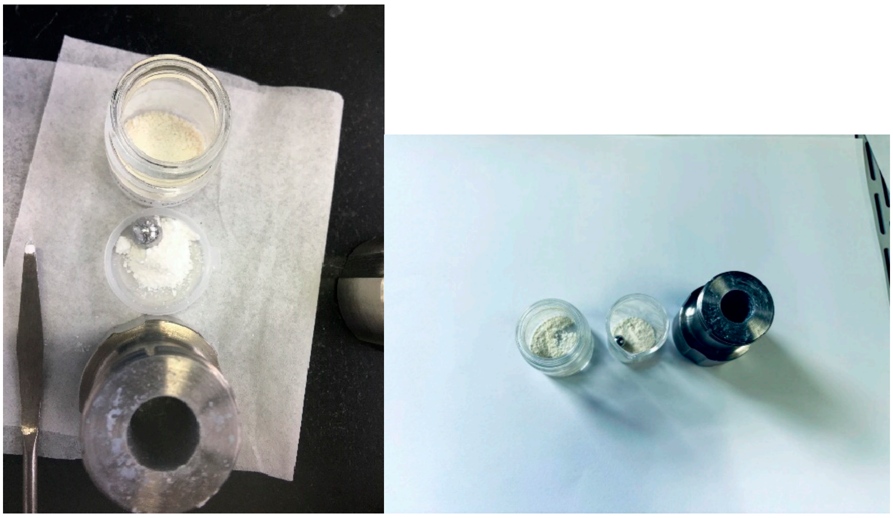

Figure S3. Photo of the mixture of reactants after LAG: (a) left: without pre-mixing step; and (b) right: with pre-mixing step.

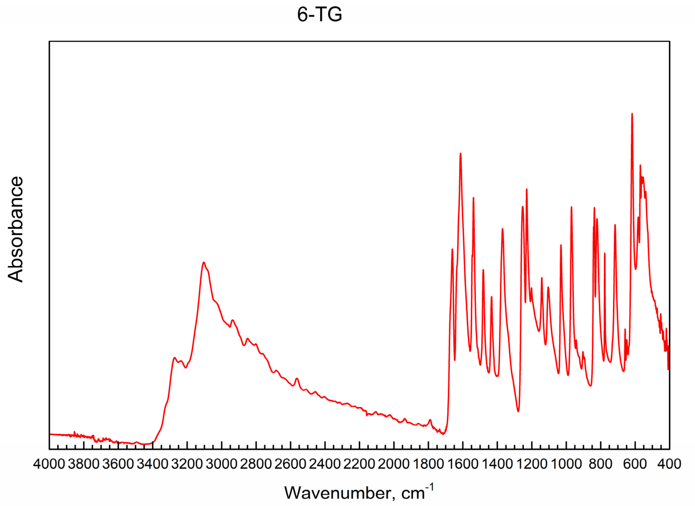

Figure S4. Survey ATR-FTIR spectrum of 6-TG.

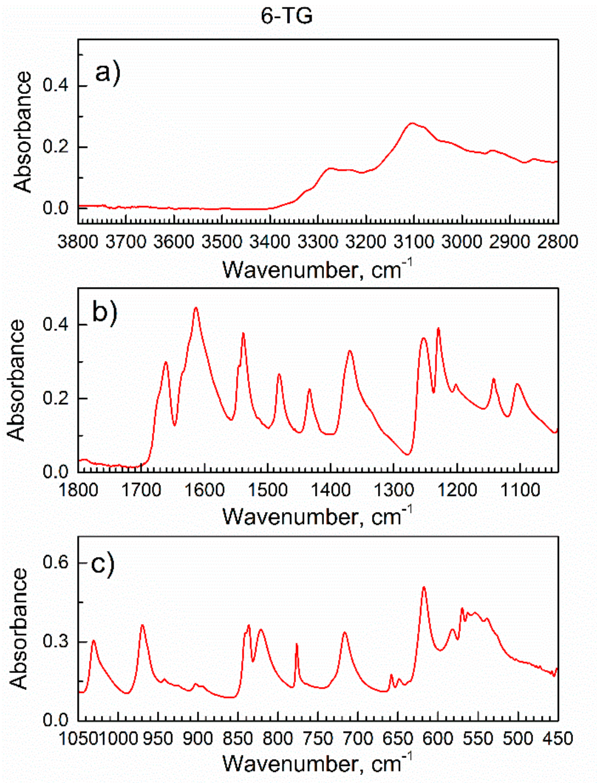

Figure S5. The ATR-FTIR spectra of 6-TG: (a) the high wavenumbers range; (b) the mid-IR range; and (c) the low wavenumbers range.

Table S1. The wavenumbers of IR peaks of 6-TG with peak assignments.

Experimental wavenumber, cm^{-1}	Literature wavenumber, cm^{-1}	Vibrational mode (predominant) of the amino-thione tautomer
3275 broad	3292	AmH16 s
3106 broad	3129	AmH15 s
1661	1666	HNH sci
1634 sh	1635	C2N3 s
1614	1618	N1H be
1539	1546	N3C4 s
1482	1483	C8N9 s
1433	1436	N7H be
1439	1375	C2Am s
1252	1259	C5N7 s
1229	1231	C8N9 s
1141	1143	N1C6 s
1103	1105	NH2 ro
1031	1032	N7C8 s
969	972	NH2 ro
840	841	R5 def
836	838	R6 def
821	824	NH16 owa
776	777	R5 ode2
716	719	N1H owa
617	621	R5 def
570	572	R5 ode

Abbreviations: Am = amino group; s = stretching; sci = scissoring; sh = shoulder; be = bending; ro = rocking; def = deformation; owa = out-of-plane wagging; ode = out-of-plane deformation.

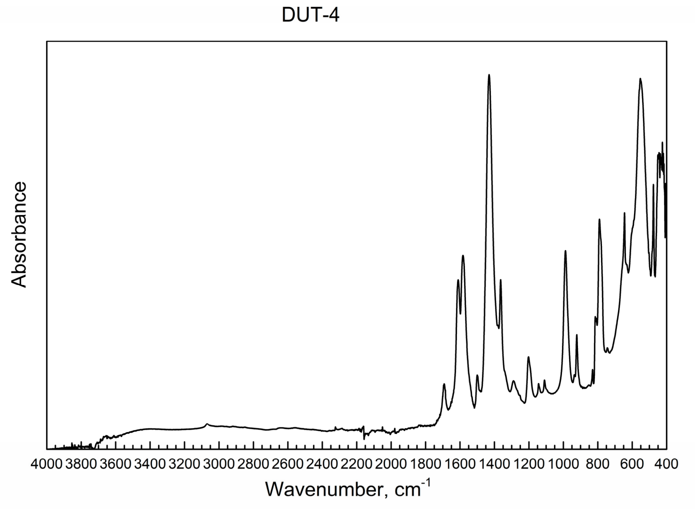

Figure S6. The survey ATR-FTIR spectrum of activated DUT-4 as matrix.

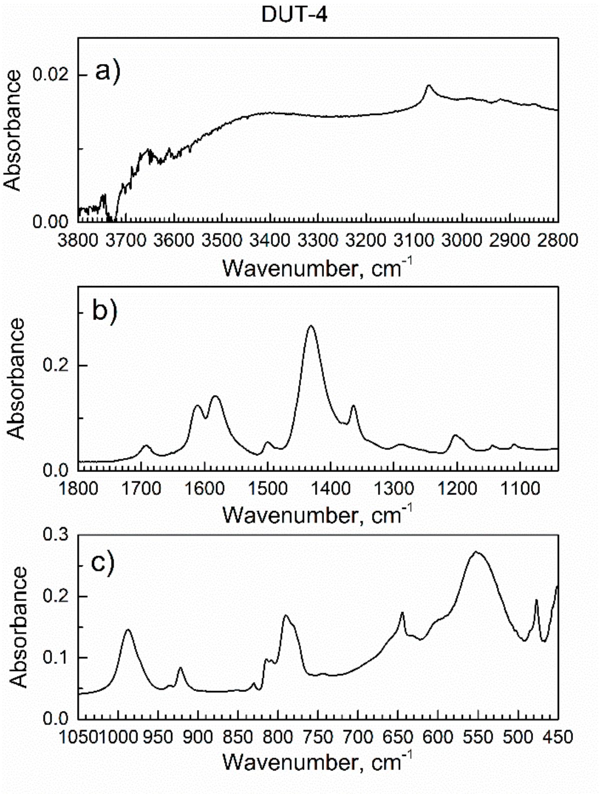

Figure S7. The ATR-FTIR spectra of DUT-4: (a) the high wavenumbers range; (b) the mid-IR range; and (c) the low wavenumbers range.

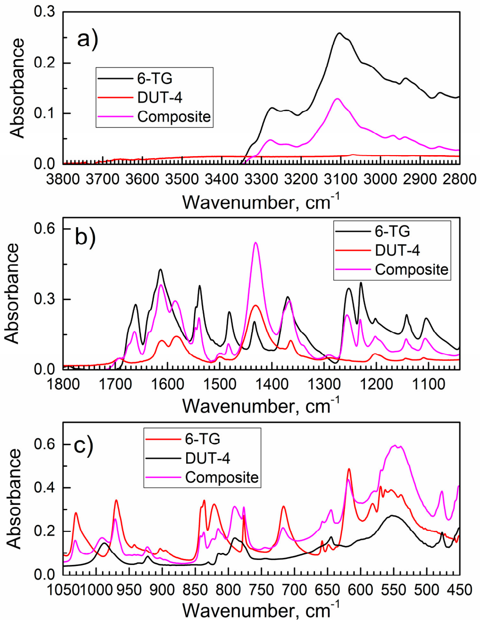

Figure S8. The ATR-FTIR spectra of 6-TG, DUT-4, and composite: (a) the high wavenumbers range; (b) the mid-IR range; and (c) the low wavenumbers range.

3.2. Delayed Release of 6-TG from Powder of Composite Versus Pure 6-TG to PBS

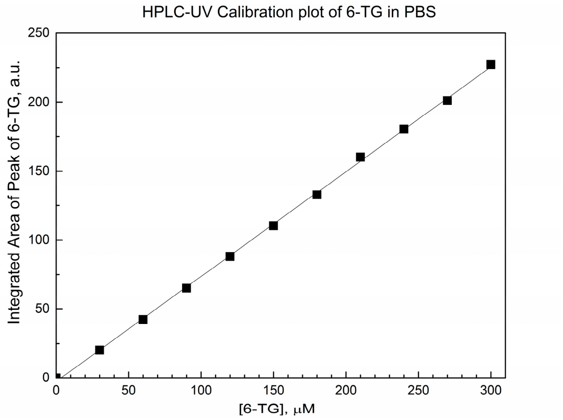

Figure S9. The HPLC-UV calibration plot of 6-TG in PBS.

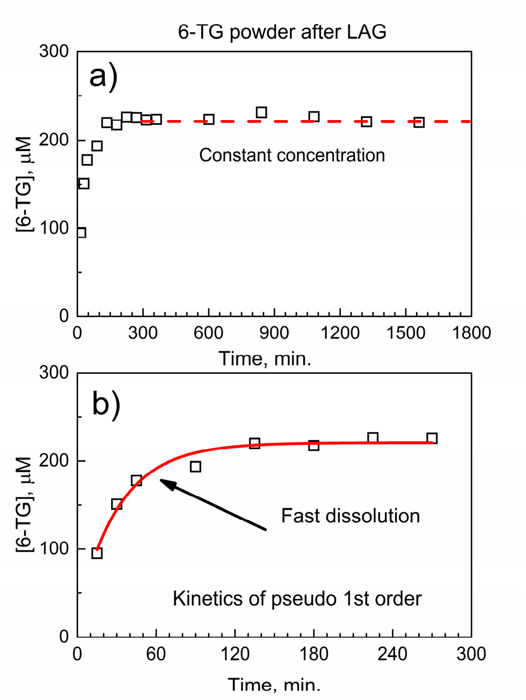

Figure S10. Temporal trace of dissolution of 6-TG powder after LAG in PBS at 37 °C: (a) drug dissolution curve; and (b) kinetic curve fitting of its initial stage.

3.3. Delayed Release of 6-TG to PBS from the Pellet at Longer Time Scale

a) 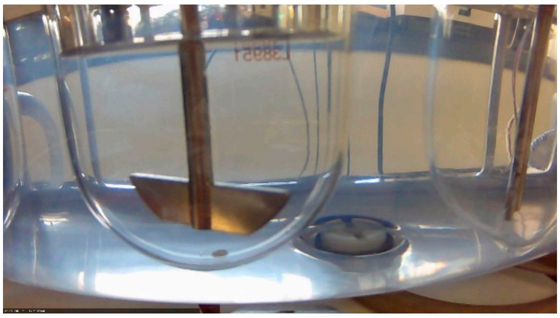

b) 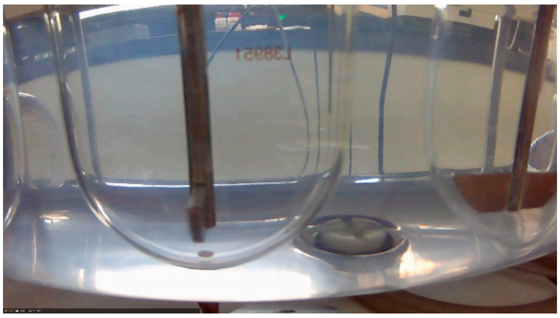

Figure S11. Photos of drug-releasing pellet in the dissolution apparatus: (a) (top) in 60 min (image # 2); and (b) (bottom) in 5820 min or 4 days (image # 194).

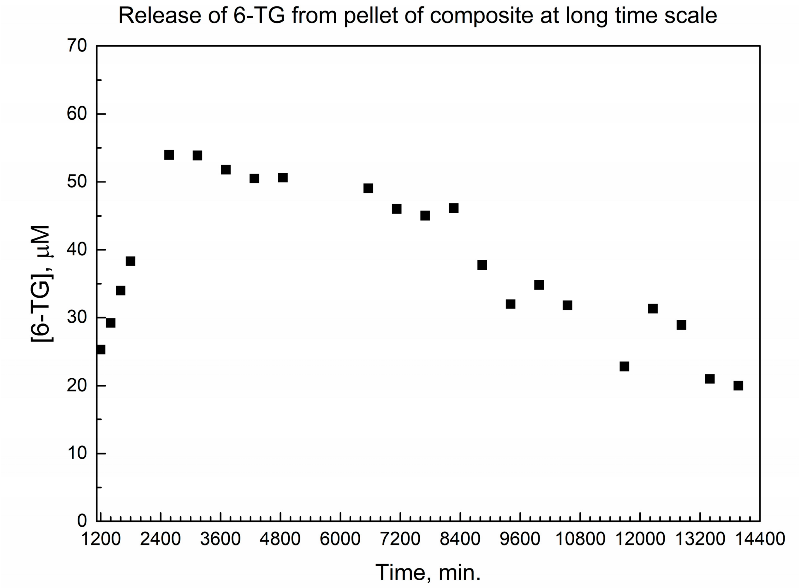

Figure S12. Extended temporal trace of delayed release of 6-TG from pellet of composite to PBS at 37 °C up to 10 days.

3.4. The In Vitro Clonogenic Assay with MV-4-11 Leukemia Cells

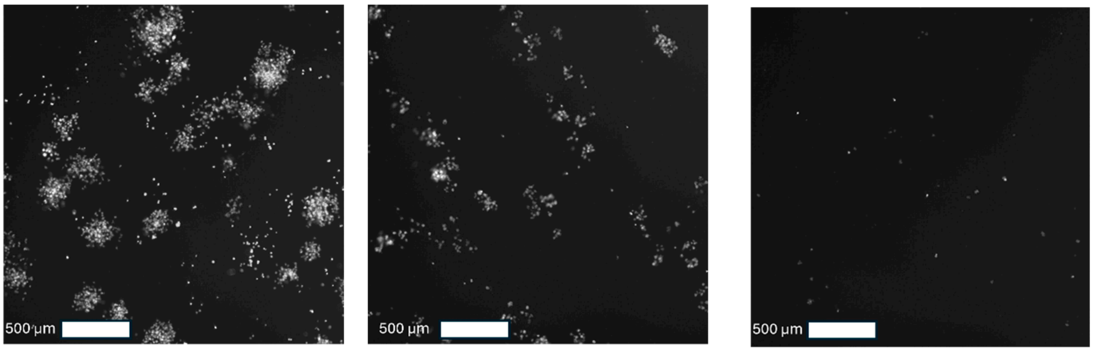

Figure S13. Representative images of the treatment groups of MV-4-11 cell colonies with 6-TG: left: control; middle: 1.2 µM; and right: 6.0 µM.

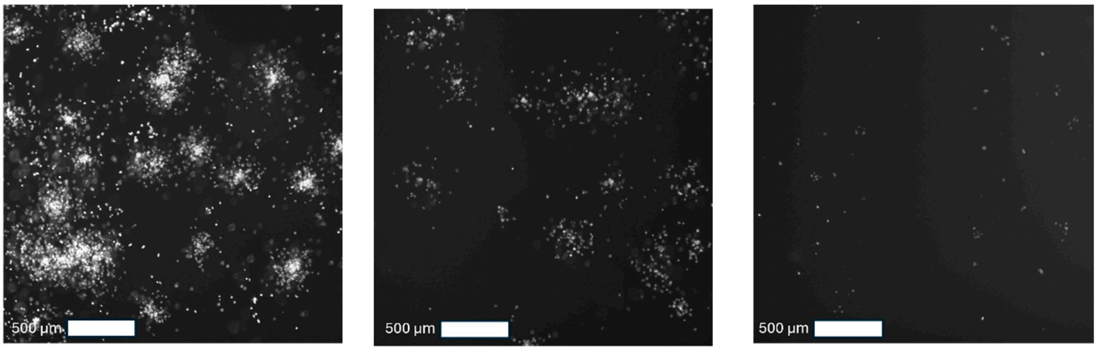

Figure S14. Representative images of the treatment groups of MV-4-11 cell colonies with composite: left: control; middle: 1.2 µM; and right: 6.0 µM.
